# Investigating the cortical effect of false positive feedback on motor learning in motor imagery based rehabilitative BCI training

**DOI:** 10.1186/s12984-025-01597-w

**Published:** 2025-03-18

**Authors:** Hojun Jeong, Minsu Song, Sung-Ho Jang, Jonghyun Kim

**Affiliations:** 1 School of Mechanical Engineering, Sungkyunkwan University, Suwon, Gyeonggi-do 16419 Republic of Korea; 2 Department of Psychology, University of British Columbia, Vancouver, V6T 1Z4 Canada; 3 Department of Physical Medicine and Rehabilitation, College of Medicine, Yeungnam University, Daegu, 42415 Republic of Korea

**Keywords:** Brain–computer interfaces, Feedback, Motor learning, Motor imagery, Event-related design, Near-infrared spectroscopy

## Abstract

**Background:**

Motor imagery-based brain–computer interface (MI-BCI) is a promising solution for neurorehabilitation. Many studies proposed that reducing false positive (FP) feedback is crucial for inducing neural plasticity by BCI technology. However, the effect of FP feedback on cortical plasticity induction during MI-BCI training is yet to be investigated.

**Objective:**

This study aims to validate the hypothesis that FP feedback affects the cortical plasticity of the user’s MI during MI-BCI training by first comparing two different asynchronous MI-BCI paradigms (with and without FP feedback), and then comparing its effectiveness with that of conventional motor learning methods (passive and active training).

**Methods:**

Twelve healthy volunteers and four patients with stroke participated in the study. We implemented two electroencephalogram-driven asynchronous MI-BCI systems with different feedback conditions. The feedback was provided by a hand exoskeleton robot performing hand open/close task. We assessed the hemodynamic responses in two different feedback conditions and compared them with two conventional motor learning methods using functional near-infrared spectroscopy with an event-related design. The cortical effects of FP feedback were analyzed in different paradigms, as well as in the same paradigm via statistical analysis.

**Results:**

The MI-BCI without FP feedback paradigm induced higher cortical activation in MI, focusing on the contralateral motor area, compared to the paradigm with FP feedback. Additionally, within the same paradigm providing FP feedback, the task period immediately following FP feedback elicited a lower hemodynamic response in the channel located over the contralateral motor area compared to the MI-BCI paradigm without FP feedback (*p* = 0.021 for healthy people; *p* = 0.079 for people with stroke). In contrast, task trials where there was no FP feedback just before showed a higher hemodynamic response, similar to the MI-BCI paradigm without FP feedback (*p* = 0.099 for healthy people, *p* = 0.084 for people with stroke).

**Conclusions:**

FP feedback reduced cortical activation for the users during MI-BCI training, suggesting a potential negative effect on cortical plasticity. Therefore, minimizing FP feedback may enhance the effectiveness of rehabilitative MI-BCI training by promoting stronger cortical activation and plasticity, particularly in the contralateral motor area.

## Background

Cortical plasticity, also known as neuroplasticity, is the brain’s ability to heal itself after an injury, enabling patients to regain brain function including motor skills [1]. This occurs through new neural connections, reorganization of pathways, and sometimes new neuron generation [2, 3]. Broadly, conventional motor learning techniques used to induce cortical plasticity after a stroke can be categorized into active and passive training methods. Passive training is beneficial in patients who are unable to move their bodies voluntarily; however, continuous repetitive movements devoid of the user’s intention are problematic. Passive training, while crucial for muscle maintenance, lacks the motor planning and cognitive engagement necessary to stimulate neural plasticity, leading to reduced neural activation, motivation, and meaningful feedback for functional recovery [4–6]. The motor imagery-based brain–computer interface (MI-BCI) is a novel solution to this problem; it provides users with the intended movement feedback based on users’ motor intention, using the modalities such as electroencephalogram (EEG) [7–13]. Delivering well-timed and correct movement feedback is critical in making MI-BCI training analogous to active training, which is the ultimate goal of rehabilitative MI-BCI [12–14].

Typically, MI-BCI systems can be classified into synchronous and asynchronous systems. Asynchronous MI-BCI is a self-paced system in which detection is continuous, whereas synchronous MI-BCI is a cue-based technique in which detection is restricted within a predetermined time frame following the cue to attract the attention of users. Several studies have claimed that asynchronous MI-BCI systems are more closely related to activities of daily living [15, 16]; however, a major disadvantage of the asynchronous system is the possibility of false positive (FP) feedback, i.e., incorrect feedback not originating from user intention, even though false positive (FP) can be present in both synchronous and asynchronous systems [17–19]. Many studies assumed that FP feedback in MI-BCI training could undermine user training and induce negative neuroplasticity [18–21]; nevertheless, the issue remains outstanding [20].

Several attempts have been made to investigate the effects of FP feedback on MI-BCI training [21–25]. Previous studies have primarily described FP feedback in BCI systems as a factor that degrades performance and causes user frustration by triggering unintended action [21, 22, 24, 25], While FP feedback can sometimes promote greater learning effects during initial learning [22, 23]; however, these studies focused either on the FP-related potential (e.g., error-related potential [25]) or the relationship between FP feedback and BCI operation ability (e.g., accuracy and performance of BCI [22–24]). The studies primarily utilized FP feedback itself in terms of BCI performance or investigated the effect of FP feedback in the viewpoint of MI-BCI accuracy, not neuroplasticity. Hence, there is still a lack of clarity about the impact of FP feedback on inducing neuroplasticity during task training. A recent study compared the corticomotor excitabilities of several MI-BCI systems using transcranial magnetic stimulation [26]; an asynchronous FP-free MI-BCI system was implemented using the cue-based approach. The results demonstrated that higher cortical plasticity was induced using this approach compared with other MI-BCIs; however, it was unable to differentiate whether the effects were due to the cues or the FP feedback because the experiment was not designed to specifically verify the effects of FP feedback. Thus, the cortical effects of MI-BCI training must be investigated under different feedback conditions.

Cortical plasticity is directly related to motor recovery after stroke, making it a critical factor in illustrating the effectiveness of MI-BCI training for motor recovery [27–29]. Numerous studies have shown that oxygenation levels are a key indicator of this plasticity [27, 29]. To assess cortical effects, brain imaging techniques such as functional magnetic resonance imaging (fMRI) and functional near-infrared spectroscopy (fNIRS), which evaluate the hemodynamic response to neural activity, can be employed [29–37]. However, real-time fMRI scanning during MI-BCI training presents significant challenges [37]. fNIRS, which has low sensitivity to body movements and is easily combined with EEG, is the optimal method for examining the effects of MI-BCI training on motor recovery [31–36]. Based on the findings from a novel work on an event-related design using fNIRS [34], we believe it is possible to investigate the effects of FP feedback on cortical activation during MI-BCI training using fNIRS.

In this study, we used a simultaneous EEG-fNIRS measurement system. While there is neurovascular coupling between EEG and fNIRS [34, 36, 38], fNIRS-based MI-BCI systems exhibit an inherent delay due to the nature of hemodynamic responses, whereas EEG is highly sensitive to electrical noise from robotic feedback. Therefore, we used an EEG to implement the MI-BCI system and the fNIRS to measure cortical activation during MI-BCI training and validated the results by comparing the effectiveness of two different cue-based MI-BCI systems—with and without FP feedback. We hypothesized that FP feedback significantly affects cortical activation during the MI-BCI training period. Furthermore, for quantitative comparisons, the MI-BCI system was compared with two conventional motor learning methods (passive and active training).

## Methods

### Participants

Twelve healthy volunteers (six men; mean age = 21.92 ± 3.34 years) and four people with a stroke (three men; mean age: 54.75 ± 9.03 years) participated in this study. The people with a stroke were recruited from Yeungnam University Medical Center, South Korea, based on the following characteristics: (1) hemiplegia caused by stroke, (2) age < 70 years, (3) normal cognitive function, and (4) able to follow simple verbal commands and freely communicate with researchers. Table 1 presents the characteristics of the people with a stroke. All the people with stroke were right-handed; whereas ten healthy participants were right-handed, while one was left-handed and another was ambidextrous. The healthy volunteers had no history of neurological or psychiatric disorders.

All volunteers received full instructions about the experiment and voluntarily agreed to participate. The study was approved by the Institutional Review Board of Daegu Gyeongbuk Institute of Science and Technology (approval number: DGIST-170721-HR-025-08).

**Table 1:** Characteristics of people with a stroke (*n* = 4)

Volunteers	Age (years)	Sex	Time since stroke (days)	Affected side	Type of stroke	MBC	MMT (finger extensor)
P1	56	Female	98	R	Hemorrhagic	4	2-
P2	49	Male	203	R	Ischemic	1	0
P3	67	Male	77	L	Ischemic	4	3
P4	47	Male	175	R	Hemorrhagic	1	0

R: right-handed side, L: left-handed side; MBC: modified Brunnstrom classification; MMT: manual muscle testing

### Study design

**Fig. 1:**
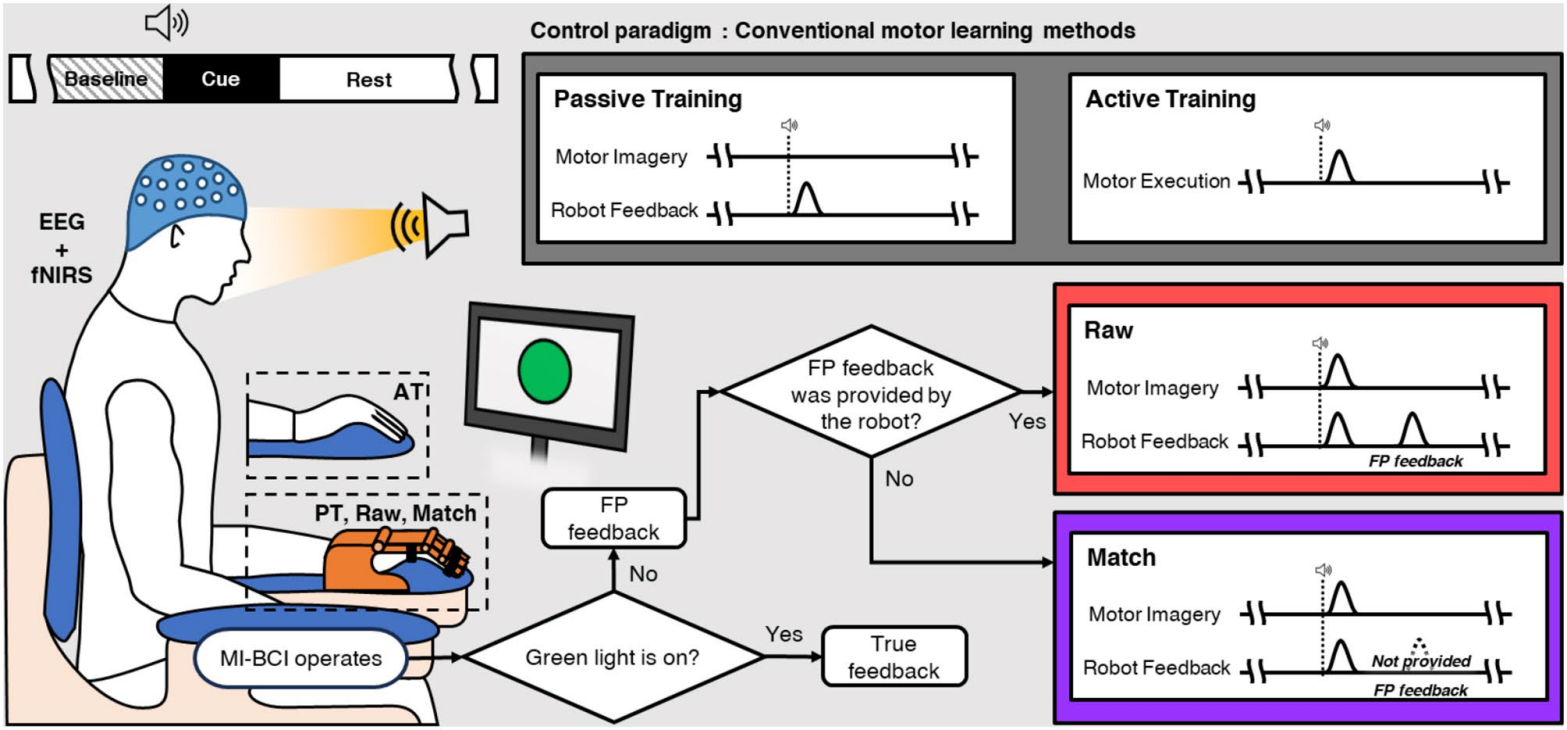
Conceptual diagram of paradigms and comparison groups (PT: passive training, AT: active training, EEG: electroencephalogram, fNIRS: functional near infrared spectroscopy)

To examine the study hypothesis, we compared the results of two MI-BCI paradigms (using asynchronous MI-BCI systems) implemented under different feedback conditions, with the Raw paradigm incorporating FP feedback and the Match paradigm without FP feedback (Fig. 1). The cued paradigm was applied to define FP clearly. In this study, FP feedback was defined as feedback resulting from a positive classification by the asynchronous MI-BCI classifier during the rest period, when the green light (cue) was off [18, 19]. Conversely, true feedback was defined as feedback from a positive classification by the asynchronous MI-BCI classifier during the task period, when the green light (cue) was on. We compared Raw and Match paradigms with two conventional methods of motor learning (active and passive training). In active training, participants voluntarily performed physical movements without any external assistance, while passive training utilized a hand exoskeleton robot that provided full assistance to participants, guiding and facilitating their movements (Fig. 1).

### Protocol

**Fig. 2:**
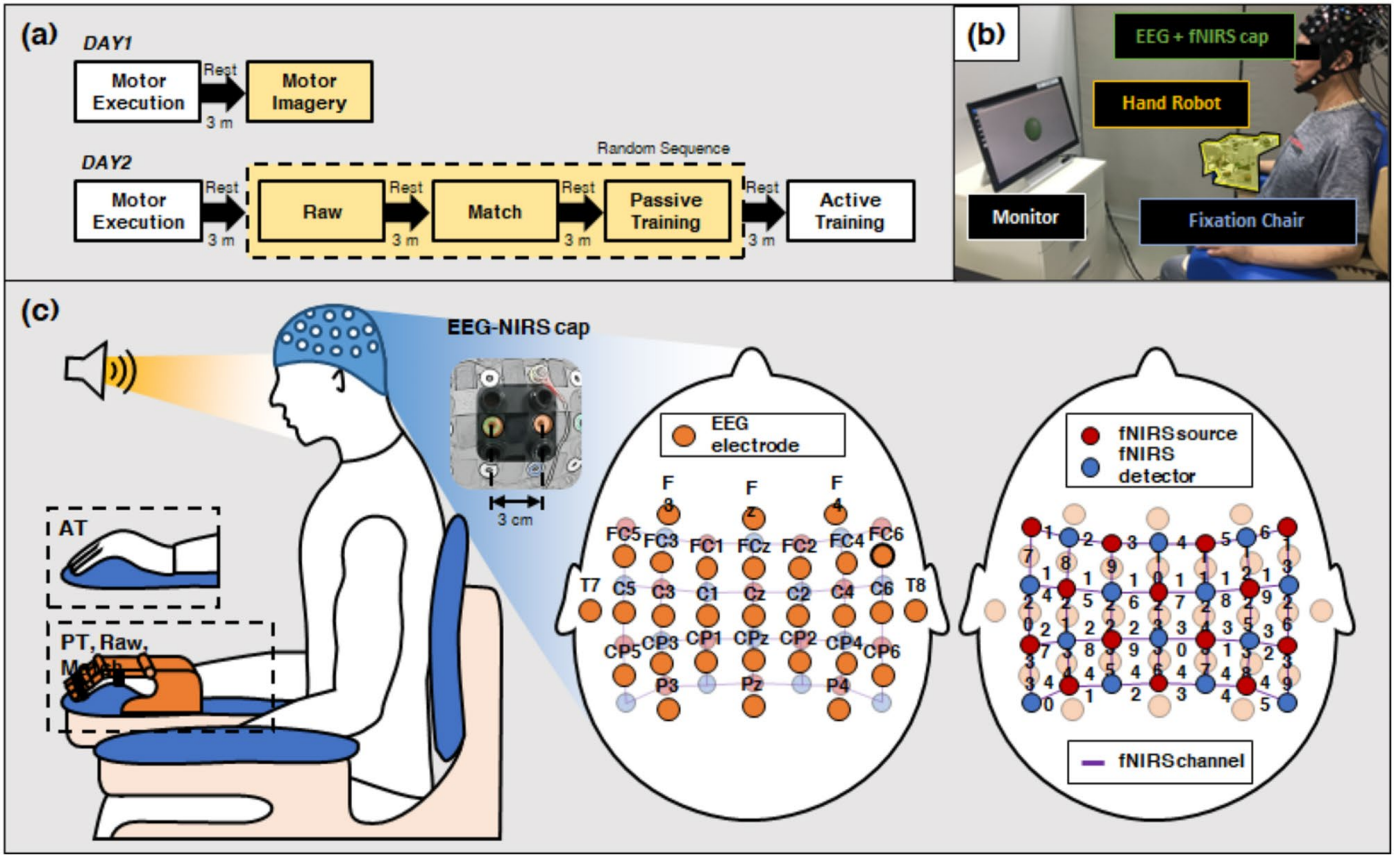
Schematic representation of the experimental protocol. (**a**) Experimental protocol; yellow boxes indicate protocols for people with a stroke (**b**) Experimental setting (**c**) Diagrammatic illustration of the placement of the EEG electrodes and the fNIRS optodes [43]

The experiment spanned two days. On day 1, motor execution and motor imagery tasks were sequentially performed to collect training data for implementing the MI-BCI (Fig. 2a). The instructed task involved performing a single open/close movement of the right hand (or the affected hand in patients with stroke) during the task. On the second day, four different paradigms (Raw paradigm, Match paradigm, active training, and passive training) were carried out; in all paradigms other than active training, movement feedback for the MI-BCI was provided through a customized hand exoskeleton robot, which provided hand opening and closing movements [39, 40]. For this experiment, we used a modified version of the MI-BCI described in previous works [18, 19]. The original MI-BCI had a two-phase classifier to minimize FP for rehabilitative BCI [18], but we modified the classifier by deleting the second phase because appropriate amount of FP is needed for this experiment. Note that all the other procedures for training and test were same to the previous works. The same experimental protocols were applied to both healthy participants and patients.

On both days, participants were seated in a chair with backrest which can fix their torso, with their right arm resting on the armrest throughout all sessions (Fig. 2b). Following a brief sound cue (lasting 0.25 s), a green light was displayed on the monitor for 2 s (or 4 s for people with stroke). Participants were specifically directed to perform the instructed task within the period when the green light was illuminated, and to relax once the green light had turned off, which lasts for 10 s (or 14 s for people with stroke). The interstimulus interval was set based on the previous studies to allow participants to sufficiently relax in the event-related design [31, 34, 41]. The sound cue for each paradigm was repeated 30 times, with 5 min of rest between each paradigm to minimize potential influence of fatigue. Any cognitive activities unrelated to the task were prevented through verbal instructions. The participants initially performed motor execution exercises to familiarize themselves with the kinesthetic sensation associated with the tasks. Following ample adaptation, passive training, Raw paradigm, and Match paradigm were randomly administered; active training was conducted last to ensure that the experience did not affect other paradigms by making the user’s MI substantially easier [34]. Patients with stroke who experienced difficulties with motor execution did not perform the task on day 1; for the same reason, they did not engage in active training on day 2 (Fig. 2a).

### Data acquisition

EEG data were recorded using the Active Two system (Biosemi, Netherlands) using the sintered Ag/AgCl electrodes on both days. A total of 29 channel electrodes were positioned on the scalp near the motor cortex, following the international 10–20 system (Fig. 2c) [42]. In addition, fNIRS data were collected alongside the EEG signals using the LABNIRS (Shimadzu, Japan) on day 2. To ensure synchronization, the digital trigger signal from the fNIRS device was transmitted to the EEG device whenever the sound cue was presented. Participants wore a head cap for EEG-NIRS simultaneous measurement (FLASH; Shimadzu Co. Ltd., Japan) [43]. The optode probe set, comprising 14 sources and 14 detectors, was embedded within the EEG electrode cap, and the neighboring source and detector pairs were spaced approximately 3 cm apart, forming a total of 45 channels (Fig. 2c) [44].

### Data analysis

EEG data from day 1 was used to train the classifier for the MI-BCI system, while the data from day 2 was employed to test the classifier, following the same methodology as outlined in the previous study [18]. Additionally, EEG data from the Match paradigm for healthy participants on day 2 was analyzed to assess brain activation during task periods with and without robotic feedback (i.e., true feedback and no feedback), in order to verify whether the MI-BCI system was functioning as expected. The EEG signals with a frequency of 2048 Hz underwent preprocessing steps, including band-pass filtering between 8 and 30 Hz and removal of independent components identified through independent component analysis, to reject the electrical noise originating from the robot [45, 46]. After epoching the EEG data (− 5 to 5 s based on the cue), abnormal epochs (> 75 µV) were rejected for noise attenuation [47–49]. A time-frequency map was visualized as an event-related spectral perturbation (ERSP) map using the EEGLAB toolbox (Swartz Center for Computational Neuroscience, USA) [50–52]; the ERSP was normalized on the baseline from − 4 to − 2 s before the cue and was displayed from − 4 to 4 s across 4–30 Hz [53, 54]. For each healthy participant, we examined the ERSP within the channel placed on the contralateral primary motor cortex (C3 for right hand) in relation to the hand motor tasks [50, 51]. In addition, to investigate the channel distribution, the averaged ERSP from 8 to 30 Hz for 2 s after the cue was calculated in each channel. ERSP and channel distribution maps were compared between two kinds of task sets, task periods with true feedback and task periods without feedback (no feedback). For statistical analysis, the averaged ERSP from the C3 channel was compared between two task sets using the Wilcoxon signed-rank test. It should be noted that EEG data was analyzed to validate whether our MI-BCI was delivering the true feedback to the users when event-related desynchronization (ERD) was detected in motor-related frequency range and area.

**Fig. 3:**
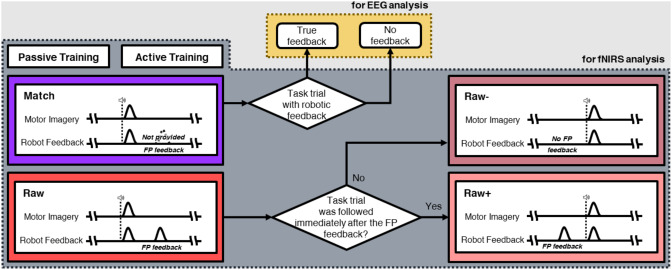
Conceptual diagram of different datasets for data analysis

fNIRS data from the four paradigms were compared to test our hypothesis about FP feedback. The Raw paradigm specifically enabled a more direct investigation of the influence of FP feedback on the user’s task periods because it included task trials right after getting feedback (Raw+) and task trials following no FP feedback in rest periods just before (Raw−) (Fig. 3). Consequently, Raw + and Raw − were extracted to explore the direct effect of FP feedback compared to the Match paradigm. The hypothesis was validated in the region of interest (ROI) channel; additionally, all channels were analyzed to determine the effect of FP feedback.

For fNIRS data analysis, we mainly used oxyhemoglobin (oxy-Hb) because it is known to be sensitive to task-related changes [55, 56]. NIRS_SPM (KAIST, South Korea), a MATLAB-based software package, was utilized for this purpose [57]. The hemodynamic data of oxy-Hb underwent preprocessing, including Gaussian smoothing (full width at a half-maximum of 2 s) and wavelet minimum description length detrending, to compensate for the noise and motion artifacts caused by the participant’s movements [58, 59]. Preprocessed hemodynamic data was segmented from − 2 to 12 s from the cue (for the data from patients, − 2 to 18 s from the cue). Baseline normalization was conducted to temporal hemodynamic response by subtracting the averaged value of the 2 s before the cue. and averaging were then applied to estimate the cortical hemodynamic response. We examined the hemodynamic responses of oxy-Hb in each channel and generated topographic distribution maps using the average hemodynamic responses within the vascular response (6–8 s after the cue) [34, 60]. Since we recruited patients with both right- and left-sided hemiplegia, the topographical distribution map for patients with left-side affected was flipped left and right to calculate the average [61]. The peak points for each paradigm were calculated in each channel and normalized by dividing them into the first motor execution task to reduce individual differences, as described in (1).

1 $$\:normalized\:peak=\frac{peak\:of\:motor\:learning\:method}{peak\:of\:motor\:execution}$$

The normalization was implemented only in healthy participants who performed motor execution tasks. For people with stroke, we cannot implement motor execution task for normalization. Since the patients’ peak values were not normalized, it was not feasible to make a direct quantitative comparison with the healthy participants’ normalized peak values.

For statistical analysis, a nonparametric Friedman test was employed to assess the ranks between parameters, followed by post hoc testing using the Wilcoxon signed-rank test as a post-hoc test [62, 63]. Most statistical analyses were conducted using IBM SPSS Statistics (version 25.0; IBM Corp, USA) [64]. After the post-hoc test for peak oxy-Hb concentration comparison, the false discovery rate (FDR) was controlled using the procedure introduced by [65], and FDR-corrected p-values were calculated with fixed lambda (0.5) in MATLAB. In the further analysis results, data from one outlier of healthy participants were excluded using the interquartile range method to ensure the accuracy of the overall tendency [24, 66, 67].

## Results

### Validation of the asynchronous MI-BCI system

**Fig. 4:**
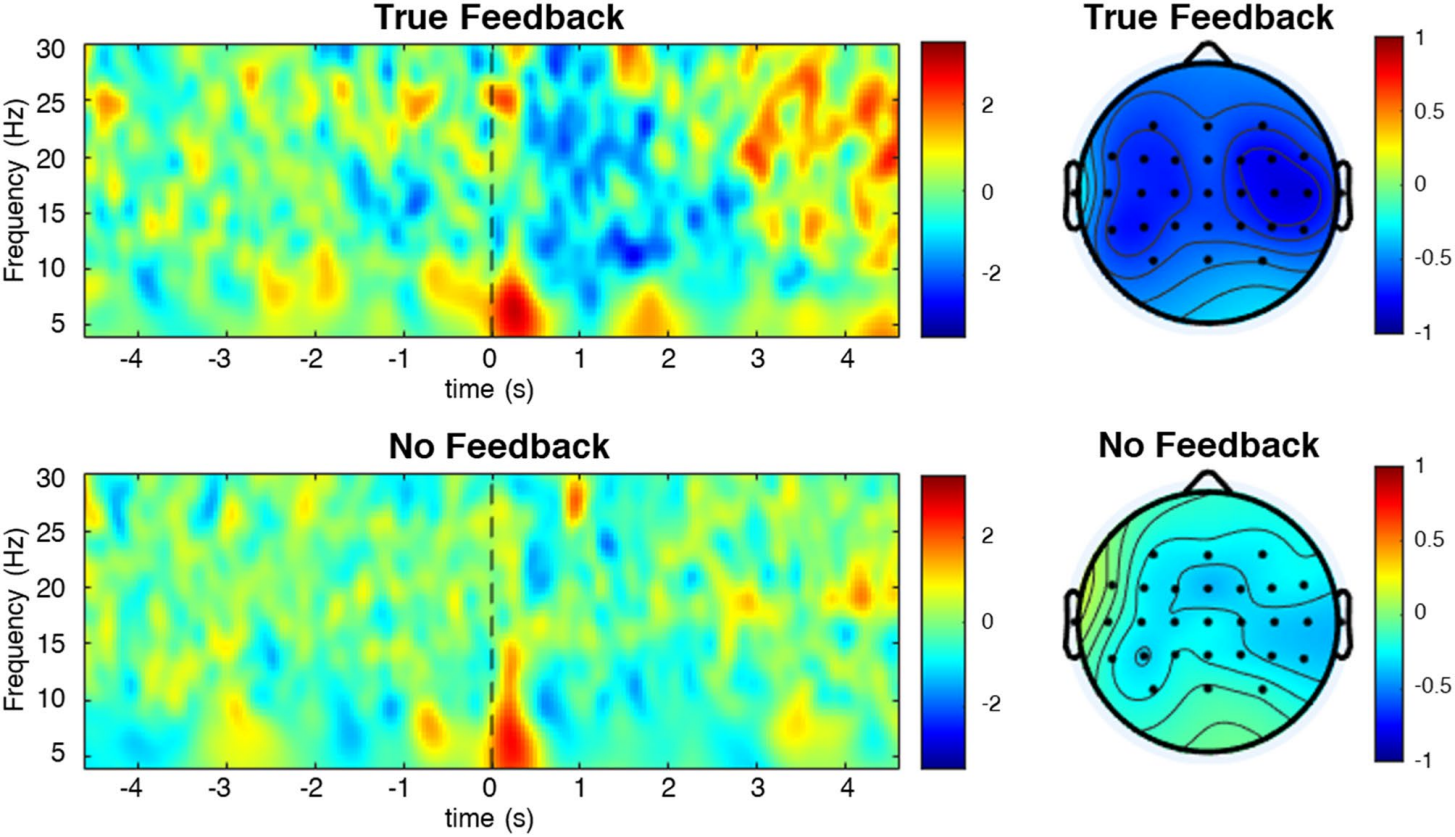
Averaged ERSP map and topographic distribution maps across all the healthy participants (dB scale). True feedback indicated the trial sets with robotic feedback for task periods, whereas no feedback indicated the trial sets without robotic feedback for task periods

Since asynchronous MI-BCI feedback was delivered to the user through a robot, we must ensure that the correct feedback is provided to support fNIRS results. Figure 4 presents the ERSP and topographic distribution maps for trials with and without true feedback used to verify whether the MI-BCI system was functioning as expected.

Trials with true feedback showed a stronger ERD of ROI channels compared with those without feedback in the mu and beta band (8–30 Hz) after the cue (*p* < 0.05). Similarly, in the topographic distribution, trials with true feedback showed a strong ERD pattern in the bilateral motor area compared to a weak ERD pattern for trials without feedback. The number of true feedback between Raw and Match paradigms was compared to guarantee a fair interparadigm comparison, and there was no between-group difference (*p* > 0.05).

### Match vs. raw paradigms

**Fig. 5:**
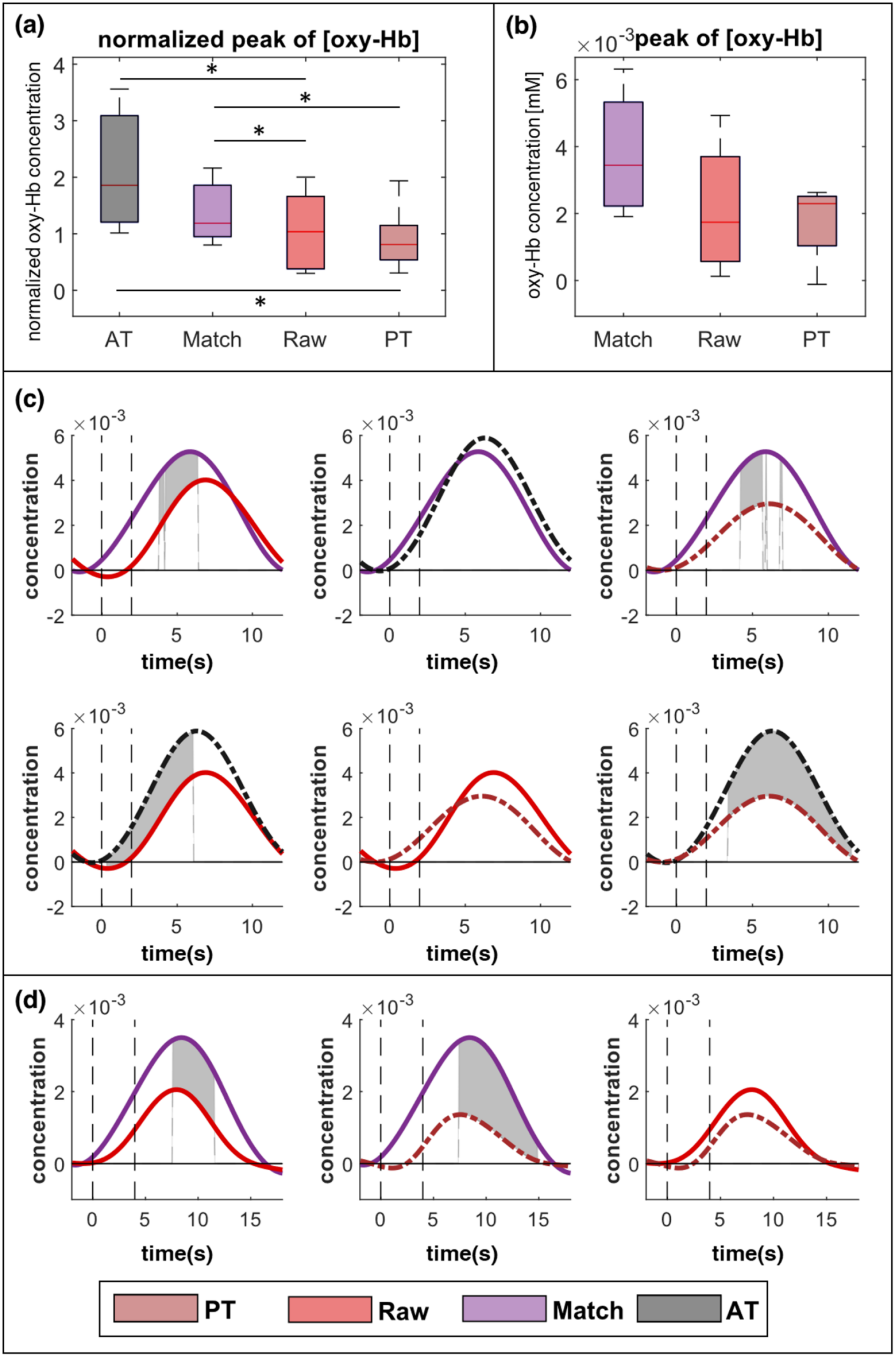
Results of the analysis of hemodynamic response between paradigms in the region of interest (ROI) channel for all study participants (PT: passive training, AT: active training). The data represent the mean values of each index, where the indices from 30 trials per participant were averaged. (**a**, **b**) Boxplots for the peak oxyhemoglobin (oxy-Hb) between each paradigm; *indicate statistically significant differences (*p* < 0.05) for the normalized peak oxy-Hb in (**a**) healthy volunteers and (**b**) patients with stroke. (**c**, **d**) Comparison of grand mean across all participants and statistical results for oxy-Hb concentrations between each two paradigms; for (**c**) healthy volunteers and (**d**) patients with stroke (red solid lines: Raw paradigm; purple solid lines: Match paradigm; black dash-dot lines: AT; brown dash-dot lines: PT). The gray-shaded area indicates a statistically significant difference in the concentrations between two different paradigms (*p < 0.05)

To investigate the effect of FP feedback during MI-BCI training, the Raw and Match paradigms were compared with the conventional passive and active training motor learning methods in the ROI channel. Figure 5 illustrates the hemodynamics in the ROI channel for all participants.

Figure 5a depicts the extent to which a paradigm elicited the maximum hemodynamic response based on the primary motor execution task. For oxy-Hb, there were statistically significant differences among the four paradigms (*p* = 0.012); post hoc testing revealed significant differences between active and passive training (*p* = 0.019), active training and the Raw paradigm (*p* = 0.029), Match paradigm and passive training (*p* = 0.038), and Match paradigm and Raw paradigm (*p* = 0.038). In Fig. 5c and d, solid lines represent hemodynamic responses for all participants.

Among healthy volunteers, active training induced the strongest average activation, followed by the Match paradigm, Raw paradigm, and passive training (Fig. 5a). In contrast, for people with a stroke, the Match paradigm elicited the highest peak of oxy-Hb concentration, followed by the Raw paradigm, and passive training; however, there were no statistically significant differences between the paradigms (*p* = 0.174; Fig. 5b). Owing to the event-related design of this experiment, statistical analysis could be performed on a time sample between different paradigms. As shown in Fig. 5c, significant differences were observed for healthy volunteers between passive and active training, active training and the Raw paradigm, the Match and Raw paradigms, and the Match paradigm and passive training. For people with stroke (Fig. 5d), the Match paradigm showed statistically significant differences from the Raw paradigm and passive training.

**Fig. 6:**
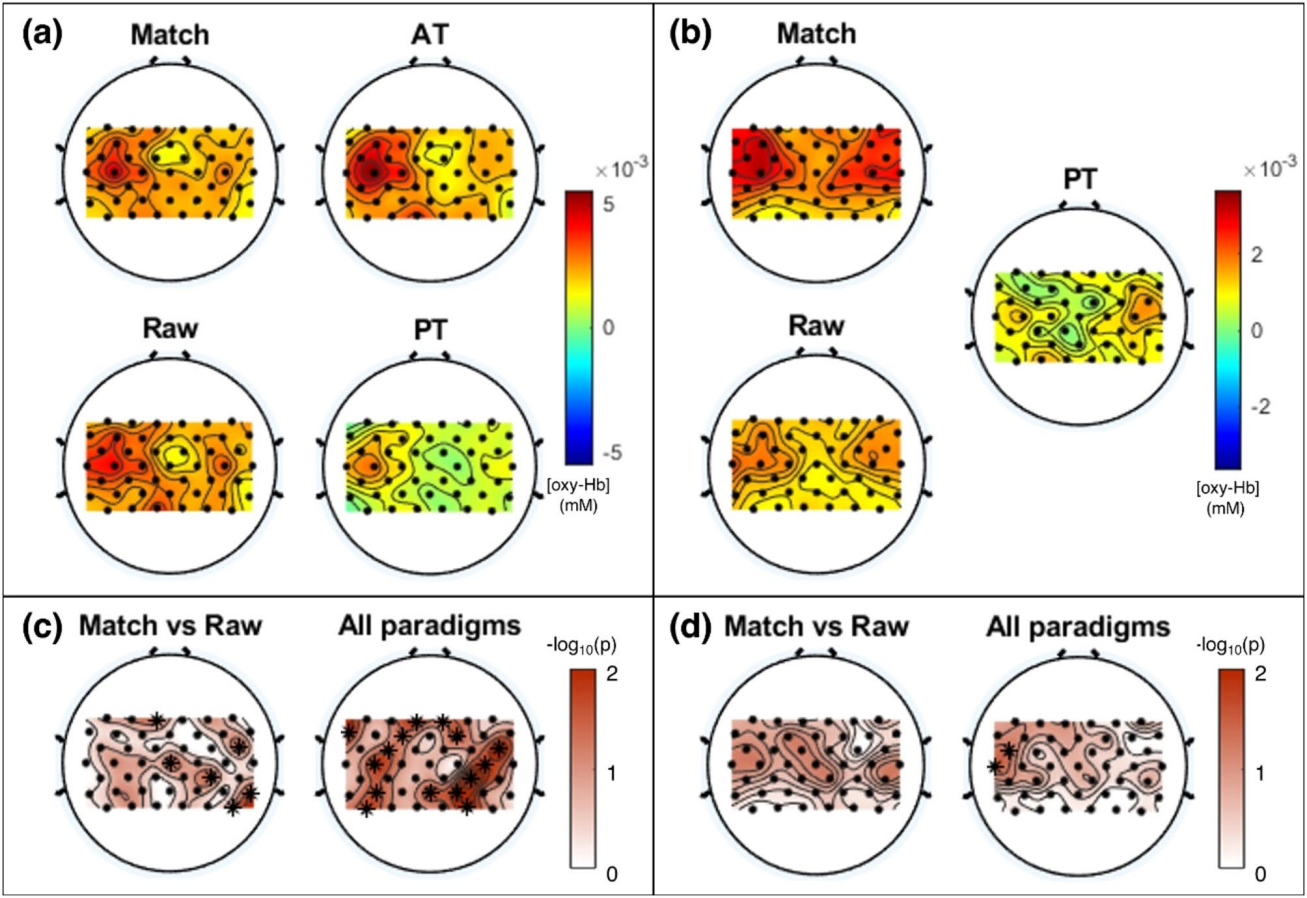
Results of the analysis of hemodynamic responses in all channels. (**a**, **b**) Topographic distribution of the average oxyhemoglobin (oxy-Hb) for different paradigms in (**a**) healthy participants and (**b**) patients with stroke (PT: passive training, AT: active training) (**c**, **d**) Topographical distribution of results of statistical comparison between all paradigms and between Match and Raw paradigms for (**c**) healthy volunteers and (**d**) patients with stroke. The color axis indicates a − log_10_(p-value); channels marked with an asterisk have a p-value < 0.05

Apart from the ROI channel, the hemodynamic response was calculated and compared for all other channels as well. In healthy participants, strong activation for oxy-Hb was observed near C3 in all paradigms (Fig. 6a and b). The overall cortical activation, including C3, was highest during active training, followed by the Match paradigm, the Raw paradigm, and passive training. Further statistical analysis was done to compare the hemodynamic response peaks across different paradigms. Figure 6c and d illustrate the topographical distribution of the statistical results obtained through the Friedman test for all paradigms and the post hoc analysis between the Raw and Match paradigms.

Healthy volunteers exhibited significantly different oxy-Hb concentrations in bilateral brain areas among all paradigms (channels 3, 4, 7, 9, 11, 15, 19, 21, 25, 31, 34, 36, 37, 40, and 44; *p* < 0.05; Fig. 6c). Among these, three channels showed statistically significant differences in the Match and Raw paradigms (channels 3, 19, and 31; *p* < 0.05). Conversely, people with a stroke had statistically significant differences in the contralateral area among all paradigms (channels 14 and 20; *p* < 0.05). There were no statistically significant differences for any channel between the Match and Raw paradigms; however, in the motor area, people with a stroke had relatively high significance between hemodynamic responses of the Match and Raw paradigms compared to other channels (Fig. 6d).

### Raw + vs. Raw

**Fig. 7:**
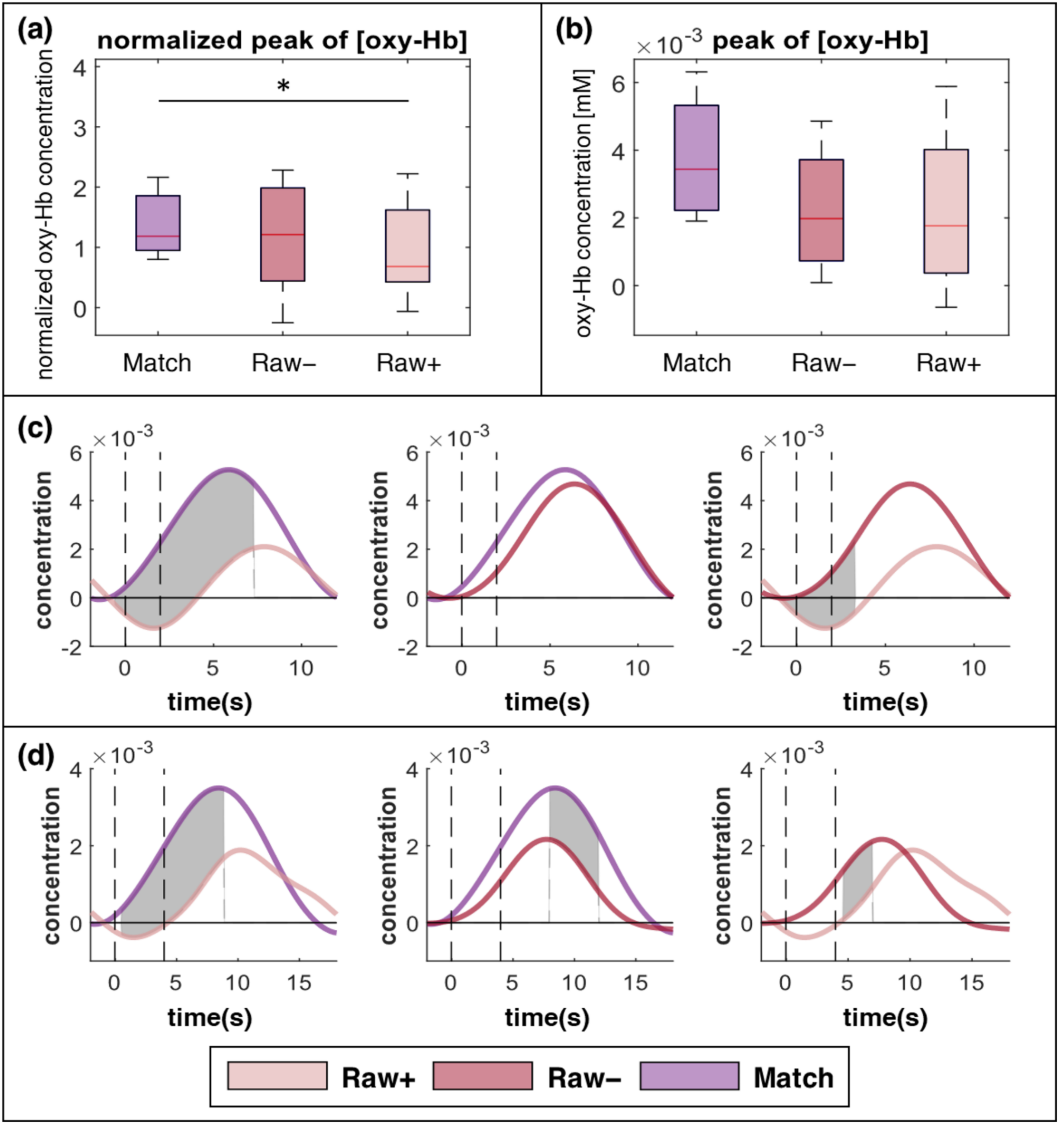
Results of the analysis of hemodynamic response between the Raw+, Raw–, and Match paradigms in the region of interest (ROI) channel for study participants. The data represent the mean values of each index, calculated by averaging the indices from all trials per participant (**a**, **b**) Boxplots for normalized peak oxyhemoglobin (oxy-Hb) between the three paradigms for (**a**) healthy volunteers and (**b**) patients with stroke; *indicate statistically significant differences (*p* < 0.05). (**c**, **d**) Comparison of grand mean and time sample data for oxy-Hb concentrations between two paradigms for (**c**) healthy volunteers and (**d**) patients with stroke (dark red solid lines: Raw+; pink solid lines: Raw–; purple solid lines: Match paradigm). The gray-shaded area indicates a statistically significant difference in the concentrations between the two paradigms (*p < 0.05)

The trial sets Raw + and Raw– were extracted from the Raw paradigms and compared to investigate the effectiveness of FP feedback (Fig. 7). For healthy volunteers, the normalized peak oxy-Hb concentration showed that Raw + had significantly lower activation than the Match paradigm (*p* = 0.022), whereas there were no significant differences between the Raw − and Match paradigms (*p* = 0.099), and Raw − and Raw+ (*p* = 0.116; Fig. 7a). For people with a stroke, the peak oxy-Hb concentration was not significantly different between the Match paradigm and both Raw+ (*p* = 0.079) and Raw− (*p* = 0.084) (Fig. 7b). Further statistical analysis using time samples revealed that healthy volunteers had statistically significant differences between the Match paradigm and Raw+, and the early phase of Raw + and Raw− (Fig. 7c). In contrast, in people with stroke, the Match paradigm showed a higher hemodynamic response than the early phase Raw + and Raw − in periods after the peak (Fig. 7d). Raw − showed higher activation than Raw + in the early phase.

**Fig. 8:**
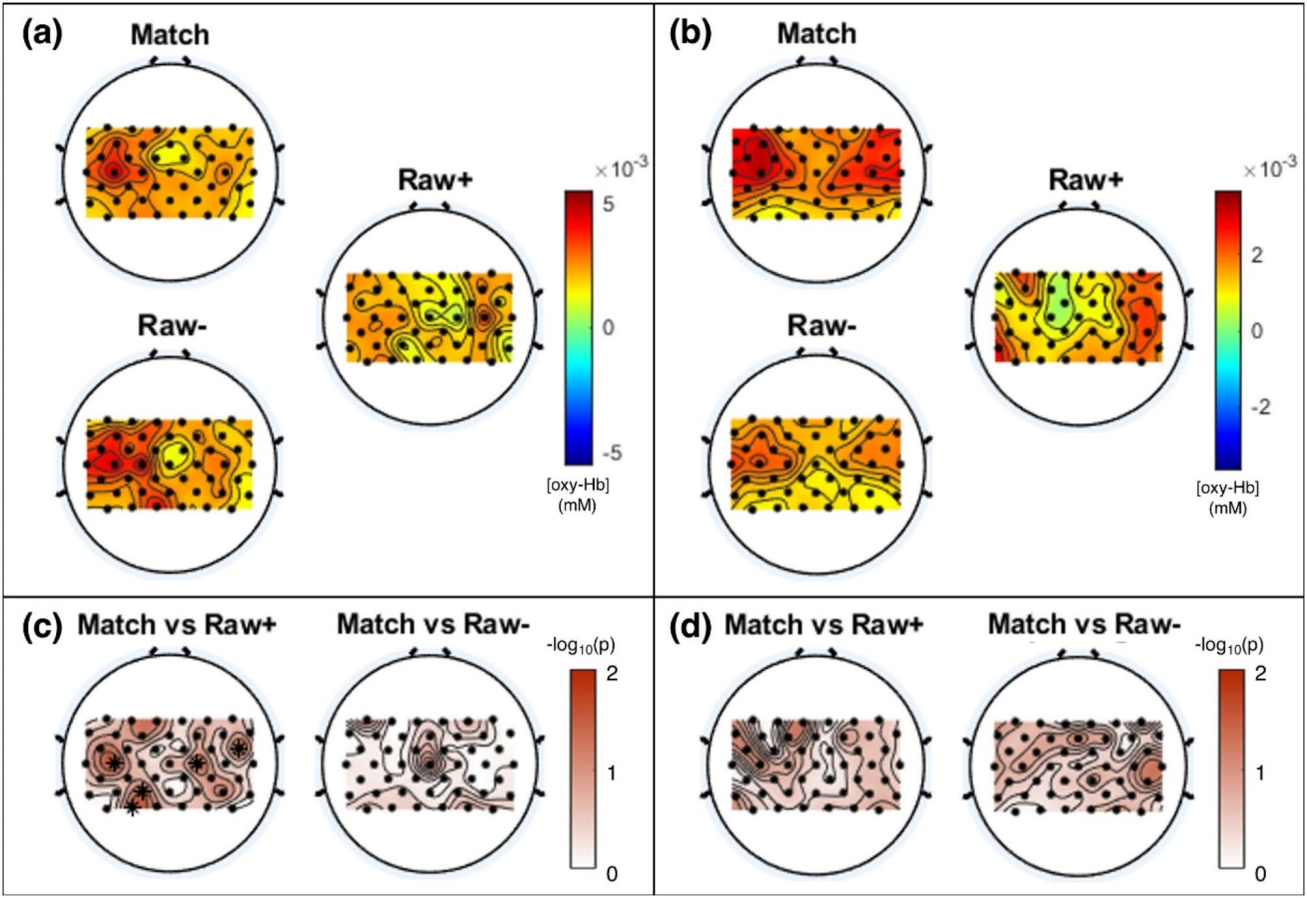
Results of the comparison between the Raw+, Raw–, and Match paradigms for all channels. (**a**, **b**) Topographic distribution for different paradigms from (**a**) healthy volunteers and (**b**) patients with stroke (**c**, **d**) Topographical distribution of results of statistical analysis between the three paradigms for (**c**) healthy volunteers and (**d**) patients with stroke. The color axis indicates the -log_10_(p-value); the channels marked with an asterisk showed statistically significant differences (*p* < 0.05)

Figure 8 depicts the comparison results for all the channels between the Match paradigm and the Raw + and Raw–. For healthy volunteers, the Match and Raw– showed a higher hemodynamic response in the contralateral area, whereas Raw + showed weak overall activation compared to the Match paradigms and Raw– (Fig. 8a). Furthermore, Raw + showed significantly lower activation than the Match paradigm in channels 19, 21, 24, 35, and 41 (*p < 0.05), while there were no significantly different channels between the Raw– and Match paradigms (Fig. 8c). In contrast, people with stroke showed bilateral activation in the Raw– and Match paradigms, with stronger activation in the Match paradigm compared to Raw– (Fig. 8b). Raw + showed strong activation in the ipsilateral motor area compared to Raw– but not in the contralateral area. There were no significantly different channels between the Match paradigm and the two Raw paradigm trial sets (Fig. 8d).

## Discussion

This study investigates whether FP feedback affects the user’s motor learning during rehabilitative MI-BCI training. We used asynchronous MI-BCI paradigms with and without FP feedback (Raw and Match paradigms), along with two motor learning paradigms of conventional rehabilitation training. Additionally, the trials after the rest periods with and without FP feedback (Raw + and Raw–) were extracted separately from the MI-BCI with FP feedback (Raw) to demonstrate the effect of FP feedback in the same paradigm and compare it with the MI-BCI paradigm without FP feedback (Match). To ensure adequate feedback of the asynchronous MI-BCI implemented in this study, we compared the EEG results in the task trials with true robot feedback and without robot feedback from Match paradigm. A mu and beta ERD pattern after the sound cue was shown in the ERSP map when the true feedback was provided, whereas a weak ERD pattern appeared in no feedback trials. According to previous studies, mu and beta ERD pattern in bilateral activation indicated the hand MI related signal [34, 50, 51]. Considering the activation occurring immediately after the sound cue to represent the activation originating from the participants’ MI, we believe that the asynchronous MI-BCI sufficiently reflected the participant’s movement intention.

To validate the effect of FP feedback on cortical plasticity induction, we compared the Raw and Match paradigms, of which the Raw paradigm tended to induce significantly lower cortical activation compared to active training. In contrast, no significant difference was observed between the Match paradigm and active training, implying that training without FP feedback had similar cortical activation as active training, which is notably advantageous for inducing neuroplasticity. In addition, the Match and Raw paradigms showed significant difference in normalized peak comparison, and the Match paradigm induced a significantly higher oxy-Hb concentration than the Raw paradigm in the 4–6 s period after the cue. Considering that Match paradigm showed higher peak in average than Raw paradigm, Match paradigm can induce earlier and more time-locked response compared to Raw paradigm. The people with stroke in this study also demonstrated a similar tendency for hemodynamic response in the ROI as healthy volunteers; however, we could not compare the two MI-BCI paradigms with active training since some of these participants had difficulties in performing their hand movements voluntarily. Even though there were no significant differences in the peak hemodynamic response between the Match paradigm, Raw paradigm, and passive training, the Match paradigm showed significantly higher hemodynamic response in the specific time range of 8–11 s after the cue compared to the Raw paradigm and passive training. These results suggest that the Match paradigm, which induced a more precisely time-locked response, also showed higher oxy-Hb concentration near the average peak points compared to the Raw paradigm. Unlike the results observed in healthy individuals, the peak of the hemodynamic response was not delayed in stroke patients, despite the delayed response observed in the healthy group. The absence of a delayed response in the Raw paradigm for stroke patients can likely be attributed to the difference in the number of FP feedback instances provided to each group, even though efforts were made to control for the number of FP feedback. This observation led us to perform a detailed analysis of the individual conditions within the Raw paradigm (Raw + vs. Raw–) to further investigate this phenomenon.

We also conducted a comprehensive analysis of the hemodynamic response across all channels, encompassing the C3 area corresponding to the left postcentral gyrus. Irrespective of the participant’s health status, the Match paradigm consistently elicited stronger hemodynamic responses near the contralateral motor area compared to the Raw paradigm (Fig. 6). In healthy volunteers, channel distribution maps for hemodynamic response from all the channels focused on the contralateral motor cortex, with the strongest in our designated ROI (i.e., C3; channel 21); however, there were some differences between the channels in different tasks. The Match paradigm showed significantly higher peak hemodynamic response in the channels corresponding to the frontal cortex (channel 3) and the ipsilateral motor cortex (channels 19 and 31) than the Raw paradigm. It means that even though the Match paradigm couldn’t induce significantly stronger activation of the contralateral motor area, it showed significantly higher cortical activity in some areas of the ipsilateral motor and frontal cortices compared to the Raw paradigm. For all channel analyses in people with a stroke, we could not obtain statistically significant differences between Raw and Match paradigms due to the small sample size; nevertheless, the pattern was similar to healthy volunteers, with significant differences for the motor area channels among passive training and the two MI-BCI paradigms. Specifically, the differences between the three paradigms were most notable in the left primary motor cortex (channels 14 and 20) near the left postcentral gyrus. Also, all patients showed a higher peak hemodynamic response in the contralateral primary motor cortex (channels 14 and 20) adjoining the contralateral postcentral gyrus during task performance in the Match paradigm compared to the Raw paradigm. Considering a history of insult to the brain on the contralateral postcentral gyrus, it is noteworthy that asynchronous MI-BCI without FP feedback promoted neuroplasticity during task periods more than the MI-BCI with FP feedback.

Further analysis of individual trial sets in the MI-BCI with FP feedback revealed a more direct effect of FP feedback in the same paradigm. In healthy volunteers, while the Match paradigm could not show significantly higher normalized peaks in the ROI compared to the Raw–, the normalized peaks were significantly higher than the Raw+ (Fig. 7a and c). Additionally, Raw– showed a significantly higher hemodynamic response than Raw + in the early phase. These results indicate that within the same paradigm, cortical activation is more induced during MI trials without FP feedback just before, compared to MI trials immediately after FP feedback. In terms of peak hemodynamic response in people with stroke, both the Match paradigm and each trial set from the Raw paradigm, Raw + and Raw–, were comparable results within the limitations of our small sample of four stroke patients. (Fig. 7b). However, the Match paradigm had a significantly higher hemodynamic response in a specific range compared to Raw+ (1–9 s after the cue), which was comparable to healthy volunteers (Fig. 7d). Also, Raw– had significantly different hemodynamic response in 5–7 s after the cue. In contrast to the results from healthy participants, Raw– demonstrated a significantly lower hemodynamic response compared to the Match paradigm during the 8–12 s window following the cue. Raw + showed slower cortical activation compared to the other conditions, Raw– and the Match paradigm, which is consistent with the results from healthy volunteers. However, unlike in healthy participants, FP feedback attenuated the hemodynamic response throughout all time periods of the Raw paradigm in stroke patients in this study.

From the viewpoint of overall channel distribution, Raw– was more similar to the Match paradigm, and Raw + showed more chaotic distribution compared to both Raw − and Match paradigms. In healthy volunteers, Raw − was similar to the Match paradigm in terms of distribution and activation level, and there were no different channels between the Raw– and Match paradigms. Alternatively, Raw + showed a more chaotic distribution with weaker activation compared to the Match paradigm. The increased oxy-Hb concentrations in the bilateral motor cortex (channels 19, 21, and 24), including the left postcentral gyrus (channel 21), and ipsilateral parietal-occipital area (channels 35 and 41) during the Match paradigm compared to Raw + highlight the distinct neurovascular responses linked to successful right hand open/close MI [34, 68–70]. Particularly noteworthy is the activation in the left postcentral gyrus (channel 21), implying a strong correlation between this region and the effective execution of the task [34, 68, 70]. The people with a stroke in this study also showed similar results, with the Match and Raw– encouraging cortical activation in bilateral motor areas and stronger activation in the contralateral motor area, whereas Raw + encouraged cortical activation in the ipsilateral motor area. Additionally, Raw + had differences in the contralateral area compared to the Match paradigm, whereas Raw– had differences in the ipsilateral brain area. Considering the contralateral representation of motor functions in the brain, activation of the left postcentral gyrus highlights a direct correlation with improvements in the right-hand MI performance during the Match paradigm [34, 68, 70]. Furthermore, when compared with Raw+, trials with FP feedback in the last rest period induced lower and slower hemodynamic responses than the Raw paradigm. Therefore, we can presume that the previous FP feedback interfered with the user’s motor learning and resulted in not only a low and slow hemodynamic response in the ROI but also a chaotic cortical activation in the overall distribution. We expect FP feedback would be a major disturbance to cortical plasticity induction during rehabilitative BCI; hence, the overall results highlight the importance of minimizing FP feedback to induce cortical plasticity in most MI-BCI users for rehabilitation purposes.

In the data analysis, we excluded one healthy outlier from the analysis by anomaly detection using the interquartile range [24, 66, 67]. The outlier volunteer showed higher activation induced by the Raw paradigm compared with the Match paradigm, particularly with Raw + than Raw–. This volunteer showed higher hemodynamic response for asynchronous MI-BCI paradigms, especially for Raw paradigm, compared to any other healthy participants. The volunteer, who had strong activation, may have been motivated by the FP feedback and attempted a stronger MI, suggesting that motivational tendencies can vary from person to person. Previous studies have shown that FP feedback does not always interfere with the BCI operation or induce wrong-directed cortical plasticity in all participants [22–24, 26]. Thus, FP feedback typically weakly interrupts users to induce cortical plasticity. This typical correlation, also observed in the present study, emphasizes the specific impact of paradigm design, especially the intentional avoidance of FP feedback, on targeted motor task performance. Nevertheless, we must consider individual differences in the effect of FP feedback to optimize rehabilitative MI-BCI training and enhance its impact. A detailed understanding of these correlations will offer valuable insights into the neural mechanisms underlying MI tasks and highlight the potential for optimizing rehabilitative BCI paradigms for enhanced motor learning and cortical plasticity induction. According to related theories, FP feedback can distort key principles of reinforcement learning and reward-based learning [71, 72], thereby impeding effective learning processes. From the perspective of reinforcement learning, accurate feedback is crucial for proper behavior modification and the development of appropriate neural circuits [71]. However, FP feedback introduces incorrect rewards, leading users to adopt inefficient motor imagery strategies. In reward-based learning, the congruence between expected and actual rewards plays a vital role; FP feedback disrupts this reward prediction error mechanism, which in turn negatively affects dopamine pathways involved in learning [72]. Repeated exposure to such erroneous feedback can diminish user trust in the system, further impairing cortical plasticity and overall learning performance. From this viewpoint, we believe minimizing the FP feedback is a typical solution to maximize cortical plasticity induction for most MI-BCI users; however, tailoring FP feedback to individual differences can enhance the effectiveness of rehabilitative BCI training for cortical plasticity induction.

In the present study, the experiments were carefully designed to verify the effects of FP feedback on the user’s MI during MI-BCI training. As we used MI-BCI training, the possible contaminants from the experimental setup need to be attenuated for valid comparison. Regarding EEG results, we performed additional pre-processing, such as independent component analysis and abnormal epoch rejection, to attenuate the effect of the contaminants [45–48]. Moreover, the use of fNIRS in our setup offered several advantages because of its robustness against electrical noise from robotic feedback and low sensitivity to body movements with pre-processing techniques [36, 58, 59, 73]. Since the oxygenation level obtained from fNIRS is an important index of cortical plasticity [27–29], our results could appropriately show the influence of MI-BCI training on cortical plasticity. Most fNIRS studies used a block design for their experiments, which is based on the accumulated response to repeated stimuli. However, because FP feedback in MI-BCI is an unpredictable event that can be modulated when repeated, the block design was not suitable for our study [30, 31, 34]. In contrast, an event-related design can demonstrate a single stimulus response and can investigate the feedback effect independently. Despite its advantages, only a few fNIRS studies have reported results based on an event-related design in MI-BCI owing to their low significance. We recently showed that an event-related design can be applied to investigate the cortical effects of MI-BCI and different motor learning methods [34]. Several studies have investigated the effects of FP feedback on MI-BCI training. While most of these studies have primarily focused on the impact of FP feedback on BCI performance, such as accuracy, or brain activation during FP feedback events (e.g., error-related potentials), a recent study has demonstrated that an asynchronous MI-BCI paradigm without FP feedback results in greater cortical excitability compared to one incorporating FP feedback. In contrast, the study did not examine the effects of FP feedback during MI-BCI training on users because it only investigated and compared corticomotor excitability pre- and post-intervention [26]. Moreover, there were differences in the existence of cues, as well as the FP feedback between the two asynchronous BCIs used, making it difficult to associate the difference between the two paradigms solely with FP feedback. In contrast, this study applied fNIRS with event-related design to investigate motor performance during the MI-BCI training with and without FP feedback, instead of comparing the motor performance pre- and post-intervention. Furthermore, as both asynchronous MI-BCI methods were designed in cue-based systems, the difference between the two methods in this study was only the existence of FP feedback. Hence, this study can investigate the effect of FP quite clearer than previous studies.

This study had several limitations. First, our event-related design was based on a fixed cue-based paradigm, which is an approach with a fixed interstimulus interval [41]. As fixed cues can be a predictable event for some participants, a random interstimulus interval would be beneficial in reducing the influence of cue prediction by the volunteer. Second, although we attempted to reduce individual differences by normalizing the peak hemodynamic responses, we could not strictly control the speed and force of MI [74]. Future studies may aim to better regulate these individual differences by providing proper verbal and visual instructions, thereby obtaining data with minimal participants-related variations. Furthermore, we believe our results for the people with stroke are an interesting finding but it could be concluded carefully. First, our participants with stroke have age difference compared to healthy participants in this study. Some researches have shown that the BCI proficiency during MI change with age, being easier to understand and use in younger people [75, 76]. Second, we recruited chronic stroke patients, who have lesser plasticity, in comparison to acute or subacute patients, which could be affected more from a FP feedback; hence, if we could recruit acute or subacute patients, the effect of FP feedback could be exaggerate compare to our results from chronic stroke patients. Third, normalization was restricted to healthy participants, thereby making it difficult to compare peak values between healthy participants and stroke patients. Although a direct comparison is not feasible, the grand mean of the hemodynamic response (Figs. 5c and d and 7c and d) allows for an indirect assessment of the differences in oxy-Hb concentration. In addition, people with stroke cannot effectively perform MI because they tend to forget their kinesthetic sensations long after the cerebrovascular accident, thereby causing diminished brain activation. To overcome this limitation, MI-enhancing methods, i.e. such as the rubber/virtual hand illusion approach [53, 77, 78], and/or motor-learning-enhancing methods, i.e. transcutaneous auricular vagus nerve stimulation [79], may help patients imagine their motor tasks to acquire more reliable and improved MI data. Lastly, further validation with a larger, more diverse population is needed. Future research with larger sample sizes should also account for handedness as a potential factor influencing functional performance, as some evidence suggests that neuroplasticity may be more readily induced in the dominant hand compared to the non-dominant hand, even in cases of stroke [80].

## Conclusions

We found that the MI-BCI paradigm without FP feedback induced significantly higher brain activation, focusing on the contralateral motor area, for the user’s MI compared with the FP feedback paradigm. Overall results illustrated that FP feedback reduced cortical activation for users during MI-BCI training, suggesting a potential negative effect on cortical plasticity. Interestingly, these findings were not limited to healthy volunteers; similar patterns were observed in some patients who had experienced a hemorrhagic or ischemic stroke. This observation opens up new avenues for investigation, as it suggests the potential for reducing FP feedback to improve the effectiveness of rehabilitative MI-BCI training in stroke patients. While these findings were consistent across healthy participants and some patients with a stroke, the small sample size and the inclusion of only chronic stroke patients limit the generalizability of the results. Further investigations with larger and more diverse populations are needed to refine these approaches and validate their broader applicability.

## Data Availability

No datasets were generated or analysed during the current study.

## Abbreviations

MI: Motor imagery
BCI: Brain computer interface
fNIRS: Functional near infrared spectroscopy
FP: False positive
EEG: Electroencephalogram
oxy-Hb: Oxyhemoglobin
ERD: Event-related desynchronization
ERSP: Event-related spectral perturbation
